# Oxidant and Antioxidant Status in Experimental Rat Testis after Testicular Torsion/Detorsion 

**DOI:** 10.22074/ijfs.2015.4216

**Published:** 2015-04-21

**Authors:** Tatjana Cvetkovic, Jablan Stankovic, Stevo Najman, Dusica Pavlovic, Dragana Stokanovic, Slobodan Vlajkovic, Marija Dakovic-Bjelakovic, Jovana Cukuranovic, Vladimir Zivkovic, Vladisav Stefanovic

**Affiliations:** 1 Department of Biochemistry, School of Medicine, University of Nis, Nis, Serbia; 2 Clinic of Nephrology, Clinical Centre Nis, Nis, Serbia; 3 Clinic of Urology, Clinical Centre Nis, Nis, Serbia; 4 Department of Biology, School of Medicine, University of Nis, Nis, Serbia; 5 Department of Pharmacology and Toxicology, School of Medicine, University of Nis, Nis, Serbia; 6 Department of Anatomy, School of Medicine, University of Nis, Nis, Serbia; 7 School of Medicine, University of Nis, Nis, Serbia

**Keywords:** Oxidative Stress, Reperfusion Injury, Spermatic Cord Torsion, Testis, Advanced Oxidation Protein Products

## Abstract

**Background:**

The aim of this study was to determine oxidative stress (OS) parameters after testicular torsion/detorsion in adult rats.

**Materials and Methods:**

In this experimental study, male adult Wistar rats were divided into four groups, each consisting of seven animals: group I-one hour right testicular torsion with subsequent orchiectomy, group II-one hour right testicular torsion followed by detorsion, group III-unilateral right-sided orchiectomy without previous torsion and group IV-control. After 30 days, bilateral orchiectomies were performed in rats with both testes and unilateral orchiectomies in rats with single testicles. Parameters of OS were determined in testicular tissue and in plasma.

**Results:**

Plasma concentrations of advanced oxidation protein products (AOPP) and thiobarbituric acid reactive substances (TBARS) were higher (p<0.05 and p<0.01, respectively), whilst the plasma concentration of the total sulfhydryl (T-SH)-groups was lower (p<0.05) in group I compared to the control group. Group II had higher plasma concentrations of AOPP compared to group IV (p<0.05), as well as significantly increased TBARS and decreased T-SH-group levels compared to groups III (p<0.05 and p<0.01, respectively) and IV (p<0.01, for both parameters). There were significant differences in OS markers between the ipsilateral and contralateral testis, as well as significant correlations among levels of both plasma and tissue markers of OS.

**Conclusion:**

The increase in TBARS levels seen throughout the experimental period indicated that OS development was caused by ischemia/reperfusion in the testicular tissue. The oxidant-antioxidant system of the testicular tissue was altered during torsion as well as detorsion.

## Introduction

Torsion of testis and spermatic cord is characteristic for adolescent and younger males, and requires emergency treatment. The incidence is 1:4000 in males aged less than 25 years (1). Urgent surgical treatment involves orchiectomy of the torsed testis or detorsion and its preservation if, on surgical exploration, the testicle is still viable. The two most important factors that determine testicular damage are the duration and degree of spermatic cord torsion (2,3). The testis remains vulnerable to oxidative stress ( OS ) mainly due to the abundance of highly unsaturated fatty acids (4). Oxidative damage is the result of an imbalance between oxidative and antioxidative systems. It is suggested that the primary source of reactive oxygen species ( ROS ) are leukocytes that infiltrate testicular tissue, but they may originate from spermatozoa as well (5). 

Increased expression of E-selectin and various cytokines is a stimulus for neutrophil accumulation and a subsequent rapid ROS generation (6). Oxidative phosphorylation in mitochondria is impaired by ischemia that consequentially leads to a decline in the level of cellular ATP and to the preservation of mitochondrial carriers in a reduced state (7). It has been demonstrated that reperfusion of the ischemic tissue promotes generation of ROS, which arise from activation of the xanthine oxidase system in parenchymal cells or from leukocytes that penetrate into interstitial tissue (8). Therefore, the treatment by detorsion may further damage the testis. Ipsilateral testis preservation leads to ischemic-reperfusion damage of both testes, primarily due to generation of ROS. Thus, reperfusion is beneficial for the ipsilateral testis by preventing ischemia-induced apoptosis and necrosis, but at the same time it may be deleterious for the contralateral testis. Testicular tissue is extremely susceptible to oxidative damage, due to high rate metabolism and cell replication, which also affects the contralateral testis (5). This may lead to functional impairment of both testes and infertility. In more than 35% of patients the spermatogram is abnormal and up to 25% become infertile (9). Beside ROS generation, there are other various theories of the mechanisms involved in sperm damage of both testes after detorsion, such as formation of antisperm antibodies, neutrophil infiltration, and decrease in contralateral blood flow. 

ROS react with proteins, lipids, carbohydrates and nucleic acids leading to impaired cell function and apoptosis. Unfortunately both enzymatic [superoxide dismutase, glutathione peroxidase, catalase ( CAT )] and non-enzymatic [glutathione ( GSH ), antioxidative vitamins] antioxidative defenses are limited. Therefore in pathologic conditions such as prolonged testicular torsion the damages can be irreversible (10). 

The aim of this study was to determine OS parameters after testicular torsion/detorsion in plasma and both testes tissue of adult rats at 30 days after the surgical procedure. 

## Materials and Methods

### Experimental animals

We conducted an experimental study on 28 adult male Wistar rats that weighed 150-190 g. Rats were obtained from the Institute of Biomedical Investigation, Faculty of Medicine, University in Nis, Serbia. All animals were treated humanely and the Ethical Committee of School of Medicine, University of Nis, Serbia approved all animal procedures. Rats were housed in a temperature-controlled room ( 23±1˚C ) on a 12-hour light and dark cycle, with ad libitum to food and water. 

### Experimental design

We randomly divided 28 male Wistar rats into four groups. The first group of rats was subjected to one hour right testicular torsion with subsequent orchiectomy ( group I ). The second group underwent right testicular torsion that lasted one hour, followed by detorsion ( group II ). In the third group, one hour after a scrotal incision, we performed a unilateral, right-side orchiectomy without previous torsion ( group III ). The fourth group served as a control and was not submitted to any surgical procedure ( group IV ). Thirty days later, bilateral orchiectomies were performed in the rats of groups II and IV, and left orchiectomies in groups I and III. 

### Surgical procedure

All surgical procedures were performed under general anesthesia induced by an intraperitoneal one-shot injection of ketamine ( 8 mg/kg ) and xylazine ( 10 mg/kg ). The skin of the scrotal area was shaved and prepared with 10% povidone-iodine solution. A mid-scrotal longitudinal incision was performed for access to both testes. Torsion was created by twisting the right testis for 720^o^ in a counterclockwise direction and maintained by fixing the testis to the scrotum with a 6-0 nylon suture passing through the tunica albuginea and dartos. The testis was left on top of the incised region, covered with a sterile gauze pad and kept moist with normal saline while the rat was kept under continuous anesthesia. After one hour of ischemia, we removed the suture; the right testis was either untwisted and removed or replaced in the scrotum for reperfusion. After each surgical intervention, the incision was closed by suture in two layers. Rats were allowed water and food. After 30 days, the rats were anesthetized and we removed their testes for further investigations. Animals were euthanized with an intracardiac barbiturate overdose injection. 

### Analytical procedure

Venous blood from the abdominal aorta was collected, centrifuged and stored at -20˚C until assay. Tissues were separately weighed and homogenized in ten volumes of cold 0.01M Tris-HCl buffer ( pH=7.4 ) using an automatic homogenizer. The homogenates were then centrifuged at 20000 g for 15 minutes at 4˚C. Clear supernatants were used for measuring CAT activity, thiobarbituric acid reactive substances ( TBARS ) and GSH content. Tissue protein levels were also quantified at this step according to the method used by Lowry et al. (11). 

We assayed the for the level of TBARS as a measure of lipid peroxidation in plasma and tissue according to the methods of Andreeva et al. (12) and Ohkawa et al. (13). Malondialdehyde ( MDA ) reacts with TBA under acidic conditions at 95˚C, forming a pink complex that absorbs at 532 nm. 

The plasma advanced oxidation protein products ( AOPP ) assay was performed as described by Witko-Sarsat et al. (14). Each well of a 96-well microplate was filled with 200 μL of supernatant diluted at a ratio of 1:5 in PBS or chloramine-T standard solutions ( 0-100 μmol/L ). Afterwards, 10 μL of 1.16 M potassium iodide ( KI ) was added followed by 20 μL of acetic acid. The absorbance of the reaction mixture was immediately read at 340 nm in a microplate reader against a blank that contained 200 μL of PBS, 10 μL of KI, and 20 μL of acetic acid. AOPP concentrations were expressed in μmol/L of chloramine-T equivalents. 

The CAT activity was determined by the spectrophotometric method based on the ability of hydrogen peroxide to form a stable stained complex with molybdenum salts (15). 

Improved method for the determination of tissue reduced GSH was based on the formation of a color product, monitored at 412 nm after the addition of Ellman reagent ( 5, 5΄-dithiobis-2-nitrobenzoic acid ) (16). 

We determined the concentration T-SH groups in plasma by using Ellman’s reagent ( 5, 5΄-dithiobis-( 2-nitrobenzoic acid ), DTNB). Absorbance was measured at 412 nm against blank samples without DTNB and expressed as mol/L (17). 

### Statistical analysis

All data were expressed as mean and standard deviation. Statistical analysis was performed using SPSS 16.0 statistical software. Parametric group data was compared using ANOVA with Tukey post-hoc test and the Student’s t test. Correlation significance was determined according to Pearson’s coefficient. p<0.05 was considered statistically significant. 

## Results

Levels of plasma markers of OS in each experimental and control group are shown in table 1. Compared to the control group, there were higher concentrations of AOPP ( p<0.05 ) and TBARS ( p<0.01 ) whereas the concentration of T-SH-groups in plasma was lower ( p<0.05 ) in the torsion/castration group. group II ( torsion/ detorsion ) had higher plasma concentrations of AOPP compared to group IV ( p<0.05 ), as well as significantly increased TBARS and decreased T-SH-groups compared to both groups III ( p<0.05 and p<0.01, respectively ) and IV ( p<0.01, for both parameters ). 

**Table 1:** Markers of oxidative stress (OS) in rat plasma


	Group	I	II	III	IV	p

Plasma (mean±SD)
	AOPP (μmol/L)	46.88±1.58 ^a^	53.5±12.86 ^a^	37.07±15.2	35.39±5.77	0.050
	CAT (U/L)	175.13± 82.54	147.16± 101.25	245.37±57.07	237.47± 82.2	0.358
	TBARS (μmol/L)	23.38±4.22 ^b^	28.54±6.52 ^b,c^	17.6±7.67	13.67±2.73	0.004
	T-SH (μmol/L)	255.72± 71.02 ^a^	217.84± 24.21 ^b,d^	327.57±85.42	354.26± 57.77	0.006


^a^; P<0.05, ^b^; P<0.01 (vs. group IV). c; P<.0.05 and d; P<0.01 (vs. group III), AOPP; Advanced oxidation protein products, CAT; Catalase, TBARS; Thiobarbituric acid reactive substances and T-SH; Total sulfhydryl groups.

**Fig.1:**
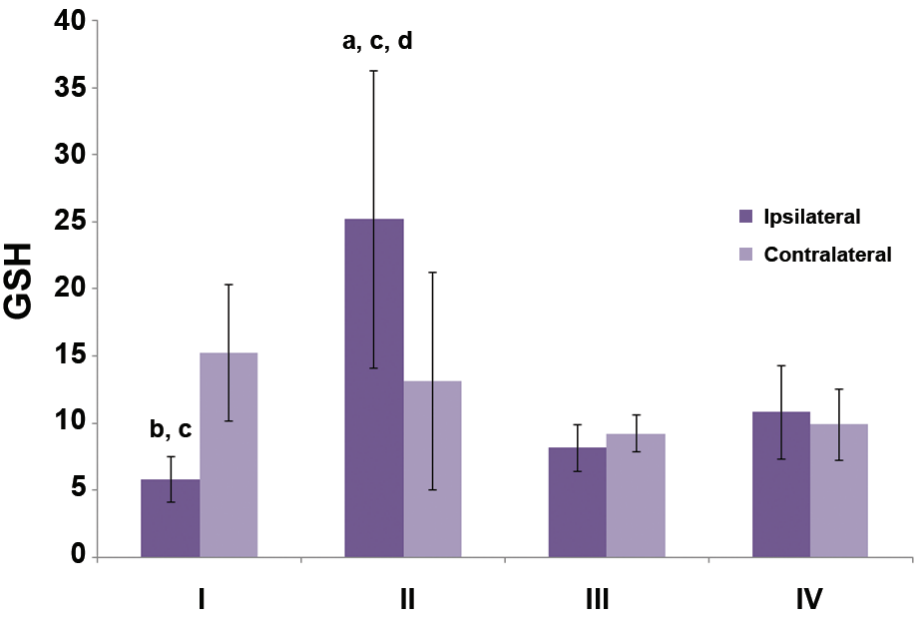
Concentration of glutathione (GSH) in testes tissues. ^a^; P<0.05 and ^b^; P<0.01 (ipsilateral vs. contralateral testis), c; P<0.01 (vs. group IV) and d; P<0.001 (vs. group III).

**Fig.2:**
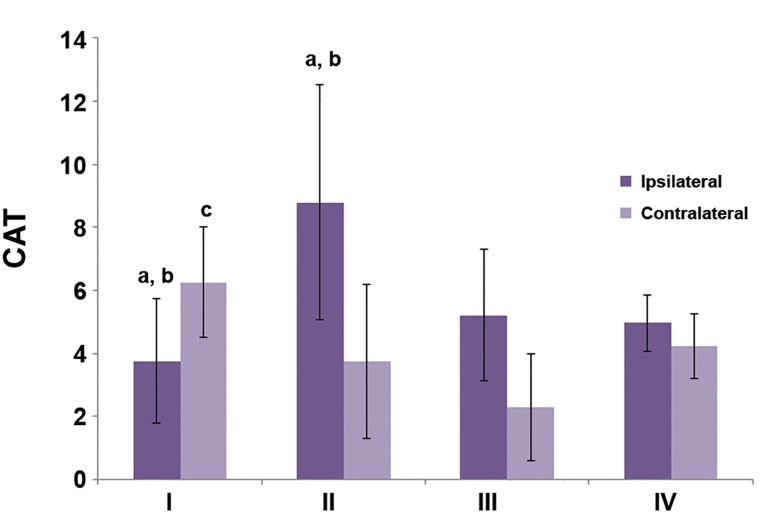
Concentration of catalase (CAT) in testes tissues. ^a^; P<0.05 (ipsilateral vs. contralateral testis), ^b^; P<0.05 (vs. group IV), c; P<0.01 (vs. group III) and d; P<0.05 (vs. group II).

**Fig.3:**
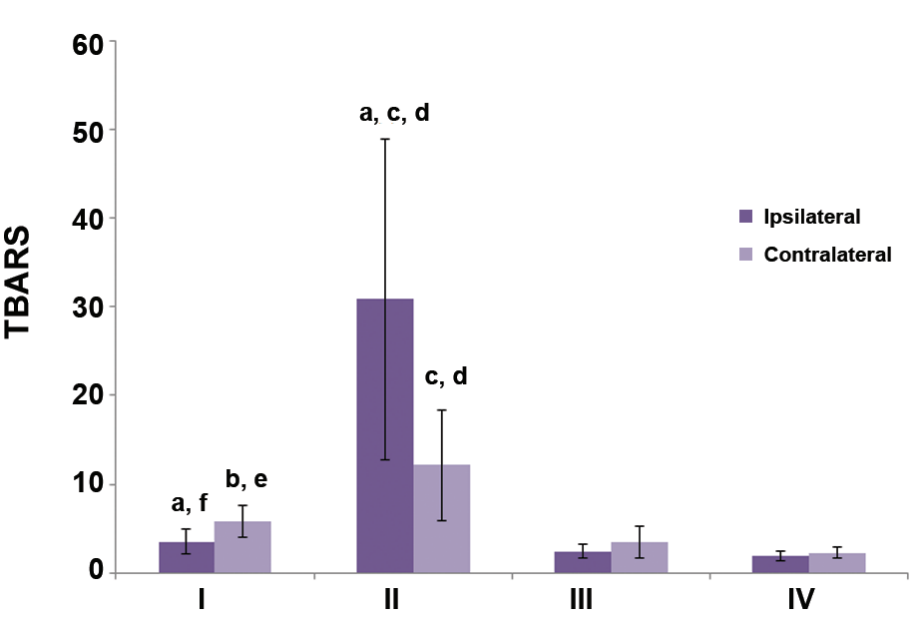
Concentration of thiobarbituric acid reactive substances (TBARS) in testes tissues. ^a^; P<0.05 (ipsilateral vs. contralateral testis), ^b^; P<0.05 and c; P<0.001 (vs. group IV), d; P<0.001 (vs. group III), e; P<0.05 and f; P<0.001 (vs. group II).

In figures 1-3, differences in markers of OS between ipsilateral and contralateral testis can be seen. There were no statistically significant differences between groups III and IV (sham operated and control rats). In group I, CAT activity was higher in the contralateral testis (p<0.05) as well as the concentrations of TBARS and GSH (p<0.01). In the torsion/detorsion group, the levels of all three markers were significantly lower in the contralateral testis (p<0.05).

We observed significantly higher CAT activity (p<0.05), TBARS concentrations (p<0.001) and GSH levels (p<0.05) in the tissue of the ipsilateral testis in group II which underwent detorsion compared to group I where the torsed testis was castrated after 1 hour. Compared to the control group, all three OS markers increased in the torsion/ detorsion group: CAT (p<0.05), TBARS (p<0.001) and GSH (p<0.01). TBARS and GSH concentrations significantly increased in the tissue of detorsed testis in group II compared to the sham operated group III (p<0.001).

Similar results were obtained for the contralateral testis. The concentration of TBARS significantly increased when detorsion was performed 1 hour after torsion, but not castration (group II vs. group I p<0.05). CAT activity increased in group I compared to group III (p<0.01). TBARS concentration was higher in group II than in both groups III and IV (p<0.001).

We observed statistically significant correlations among levels of both plasma and tissue markers of OS (Table 2). Plasma concentrations of TBARS directly correlated with plasma levels of AOPP (r=0.434, p<0.05) as well as with CAT activity in the torsed testis (r=0.427, p<0.05) and TBARS in the ipsilateral (r=0.598, p<0.01) and contralateral (r=0.595, p<0.01 contralateral) tissues of both testes. TBARS levels in the ipsilateral testis were found to be negatively correlated with the plasma concentration of the T-SH-groups (r=-0.455, p<0.05). An increase in TBARS levels in the right torsed testis was associated with increased CAT activity (r=0.567, p<0.01) and GSH content (r=0.911, p<0.001) in the same testis and TBARS levels in the contralateral testis (r=0.659, p<0.001). Similarly, we observed positive correlations between TBARS in the contralateral testis and the other markers of OS in the tissue of the same testis, CAT activity (r=0.648, p<0.001), GSH levels (r=0.597, p<0.01), as well as with plasma AOPP concentration (r=0.595, p<0.01) and GSH quantities (r=0.580, p<0.01) in the torsed testis. In both testes, ipsilateral and contralateral, two markers of antioxidative capacity (CAT, GSH) showed positive correlations (r=0.588, p<0.01; r=0.615, p<0.001, respectively). The increase in plasma AOPP concentration was associated with the increase in CAT activity in the tissue of the contralateral testis (r=0.540, p<0.01). These data showed direct correlation between plasma and tissue markers of OS in both testes.

**Table 2:** Correlation between plasma and tissue markers of oxidative stress (OS)


			Plasma				Ipsilateral testis			Contralateral testis		
			AOPP	CAT	TBARS	T-SH	CAT	TBARS	GSH	CAT	TBARS	GSH

**Plasma**	AOPP	r	-	-0.073	0.434	-0.266	0.095	0.357	0.223	0.540	0.595	0.384
		p	-	0.735	0.034	0.210	0.658	0.087	0.295	0.006	0.002	0.064
	CAT	r	-0.073	-	0.041	0.065	0.207	0.108	0.200	-0.137	0.114	-0.020
		p	0.735	-	0.850	0.761	0.333	0.616	0.348	0.522	0.595	0.925
	TBARS	r	0.434	0.041	-	-0.350	0.427	0.598	0.400	0.203	0.469	0.115
		p	0.034	0.850	-	0.094	0.037	0.002	0.053	0.340	0.021	0.592
	T-SH	r	-0.266	0.065	-0.350	-	-0.213	-0.455	-0.279	-0.063	-0.365	-0.029
		p	0.210	0.761	0.094	-	0.317	0.025	0.187	0.769	0.080	0.892
	CAT	r	0.095	0.207	0.427	-0.213	-	0.567	0.588	-0.063	0.270	-0.101
		p	0.658	0.333	0.037	0.317	-	0.004	0.002	0.769	0.201	0.640
**Ipsilateraltestis**	TBARS	r	0.357	0.108	0.598	-0.455	0.567	-	0.911	0.338	0.659	0.116
		p	0.087	0.616	0.002	0.025	0.004	-	0.000	0.106	0.000	0.591
	GSH	r	0.223	0.200	0.400	-0.279	0.588	0.911	-	0.274	0.580	0.228
		p	0.295	0.348	0.053	0.187	0.002	0.000	-	0.196	0.003	0.284
	CAT	r	0.540	-0.137	0.203	-0.063	-0.063	0.338	0.274	-	0.648	0.615
		p	0.006	0.522	0.340	0.769	0.769	0.106	0.196	-	0.001	0.001
**Contralateraltestis**	TBARS	r	0.595	0.114	0.469	-0.365	0.270	0.659	0.580	0.648	-	0.597
		p	0.002	0.595	0.021	0.080	0.201	0.000	0.003	0.001	-	0.002
	GSH	r	0.384	-0.020	0.115	-0.029	-0.101	0.116	0.228	0.615	0.597	-
		p	0.064	0.925	0.592	0.892	0.640	0.591	0.284	0.001	0.002	-


AOPP; Advanced oxidation protein products, TBARS; Thiobarbituric acid reactive substances, CAT; Catalase, T-SH; Total sulfhydryl groups and GSH; Glutathione.

## Discussion

Prolonged testicular torsion leads to testicular ischemia and high levels of free radical production (18). An increase in ROS-induced OS has been shown in testicular tissue following detorsion, indicating reperfusion injury (19). OS induces poor sperm function since mammalian spermatozoa membranes are very sensitive to oxidative damage. Leydig cell mitochondria and microsomes of testes are known to contribute significantly to an increased generation of ROS (20). Ischemia also triggers the release of cytokines causing neutrophils to infiltrate testes and may represent yet another good source of uncontrolled free radical generation for mediating the pathophysiological consequences of temporary testicular ischemia (21). Measured after different time periods of reperfusionone hour, 24 hours, 48 hours and one week, the increase in MDA levels were maintained compared to sham-operated controls (4). It has been reported that after testicular detorsion OS increases and impairs testicular functions, partially by disrupting the normal structure of seminiferous tubules and by diminishing the number of germ cells (22,23). Thus, despite testicular torsion being repaired before infarction and necrosis, there is an occurrence of I/R injury that is a classic inducer of OS. The cutoff point for the preservation of torsed testis is 12 hours. In experimental animals permanent damage occurs after 4-6 hours, but in humans it is not before 12 hours of torsion that necrosis takes place (1,9). If preserved, ischemic damage to the torsed testis is followed by reperfusion injury of both testes. In the first few hours the rapid increase in ROS generation may be compensated by natural antioxidative defenses, primarily GSH. Later, other mechanisms such as inflammation and inflammatory cell infiltration become involved and damage becomes irreversible (10). 

Biochemical markers of OS are more sensitive indicators of tissue damage and can be detected much earlier than histological alterations. The most prominent tissue alterations are due to lipid peroxidation. Even if sperm count is not significantly impaired, infertility may result from low motility or DNA damage caused by OS (24). The time of reperfusion plays an important role as a determinant of the extension of reperfusion injury in both testes. Independent of torsion time, after one hour of reperfusion the content of GSH decreases and the CAT activity and levels of TBARS begin to increase in both the ipsilateral and contralateral testis (10,25,27). After a short period of time (4,19), a month or even more than a month after detorsion (28,29), MDA levels drastically increase. The results of present study have shown that preservation of the detorsed testis increased oxidative damage in the contralateral one. There was a significant difference in TBARS concentrations between both torsion/castration ( group I ) and torsion/detorsion models ( group II ) on one side, and the sham-operated ( group III ) and control ( group IV ) groups on the other side. Additionally, lipid peroxidation was more intense in the contralateral testis when the detorsed testis was preserved compared to the model where castration was performed after one hour of ischemia. Increased levels of AOPP and TBARS, and decreased levels of T-SH-groups in plasma in both models of testicular torsion suggested that ROS generated in the torsed testis might have systemic effects including those on the contralateral testis. 

The reduced availability of cellular GSH becomes a rate limiting factor for detoxification of oxygen metabolites, most likely hydrogen peroxide and lipid hydroperoxide. Simultaneously, an important accumulation and release of oxidized GSH occurs which causes further reduction of the GSH/GSSG ratio and a shift of the cellular thiol redox state toward oxidation. Ischemia that lasts for three hours or less increases OS and diminishes antioxidative GSH to a level sufficient to disrupt spermatogenesis. In the first hours of reperfusion, the level of reduced GSH has been shown to decrease. However after 48 hours its concentration was significantly above those observed in control groups and the ischemic model (4,30,31). In other short-term torsion/detorsion models (19), GSH diminished in the ipsilateral testis, while plasma total antioxidative capacity remained unchanged. In our experiment, ischemia did not change GSH levels in either of the testes. On the other hand, reperfusion significantly increased the concentration of GSH in the ipsilateral testis, which suggested an increase in antioxidative defenses. 

In our experiment, plasma CAT activity was unchanged. Concerning testicular tissue CAT, we observed an increase in its activity in the castrated testis one hour after torsion, while in the contralateral testis there were no alterations. This result implied that in this model the contralateral testis was less affected by ischemia of the ipsilateral testis compared to the second model where detorsion was performed. In the second model, CAT activity significantly increased in the ipsilateral testis a month after detorsion due to increased ROS levels. Other studies’ results were inconclusive; some confirmed the current study results (25,30), whereas others had the opposite findings (26,29). 

The correlations found between levels of various markers of OS in both testes and plasma directly implicated a causal relationship between processes ongoing in torsed/detorsed testis and the effects on the contralateral testis. 

In this experimental study we have used adult rats with the assumption that their testes are less vulnerable to OS than testes of young rats. The second difference was the extended period of reperfusion ( 30 days ) and the evaluation of OS parameters in both plasma and testes tissues. 

Since reperfusion potentiates ROS generation and subsequent impairment of both testes, a possible solution could be ischemic preconditioning. Numerous studies with various protocols have shown diminished testicular injury (25,32). Preconditioning may also be pharmacological: various antioxidative, antiinflammatory and immunosuppressive agents have been proposed as beneficial in preserving male fertility after unilateral testicular torsion. 

## Conclusion

The increase in TBARS levels seen throughout the experimental period indicated that ischemia followed by reperfusion of testicular tissue caused OS. The oxidant-antioxidant system of the testicular tissue changed during torsion as well as detorsion. 
